# Predictors of effective clinical teaching – nursing educators’ perspective

**DOI:** 10.1186/s12912-022-00836-y

**Published:** 2022-03-07

**Authors:** O. M. Al-Rawajfah, L. Al Hadid, G. K. Madhavanprabhakaran, F. Francis, A. Khalaf

**Affiliations:** 1 College of Nursing, Sultan Qaboos University, P.O. Box 66, Al Khoud, Muscat, Oman; 2 Faculty of Nursing, Al Balqa Applied University, P.O. Box 206, Salt, 19117 Jordan; 3 Faculty of Health Sciences, Kristianstad University, Elmetorpsvägen 15, SE-291 88 Kristianstad, Sweden

**Keywords:** Effectiveness, Clinical teaching, Nurse educator, Remote teaching, Nursing education

## Abstract

**Background:**

The clinical teaching is the core component of the nursing curriculum, the alarming pandemic rates brought uncertainty to clinical teaching, weighing the safety of patients, students, and faculty, which demanded essential modification in clinical teaching and resulted in challenges in relation to effective response to clinical teaching requirements. This study aimed to assess the effective clinical teaching from the nurse educators’ perspective during the remote teaching that followed the COVID-19 pandemic.

**Methods:**

This study is a national Web-based descriptive study. Participants were recruited from five major Nursing Colleges in Oman. Descriptive and inferential as well as multiple linear regression analyses were conducted.

**Results:**

A total of 127 nurse educators completed the survey with mean age of 43.9 (SD = 6.9) years. The overall effective clinical teaching score was 54.4 (SD = 10.9) which is considered acceptable, although the nurse educators in Oman reported the highest score on the safety dimension of the effective clinical teaching. Furthermore, females, doctoral prepared nurse educators, and those who acted as preceptors reported higher effective clinical teaching levels compared to their counterparts. The regression analysis showed that age, gender, and attending infection control training are significant predictors of effective clinical teaching.

**Conclusion:**

The paradigm shift in clinical teaching requires adequate measures including identification and appropriate training of clinical instructors and preceptors to meet clinical teaching demands in remote teaching. It is also important to take actions that promote and maintain the safety prioritization in bedside clinical teaching. These measures might positively impact on the nursing education process.

**Supplementary Information:**

The online version contains supplementary material available at 10.1186/s12912-022-00836-y.

## Background

The outbreak of coronavirus disease 2019 (COVID-19) erupted in Wuhan, China in December 2019 and reached pandemic level in March 2020 [1]. According to the latest statistics published by the WHO on January 22, 2022, there were more than 323 million confirmed cases with more than 5.5 million deaths globally. In the Eastern Mediterranean region, the pandemic’s effects were also disastrous, with more than 17.5 million confirmed cases and 318,268 deaths [2]. Furthermore, the Sultanate of Oman has been greatly affected by the pandemic. With the total lockdown of societies including educational facilities, the extensive spread of the pandemic has resulted in drastic challenges and changes in nursing education including suspension of training of nursing students in clinical settings.

One of the most important challenges is the quick and effective response of nursing educators to modify the learning experience to be compatible with safety requirements for patients, students, and nursing educators [3–5]. The response to these challenges was managed at institutional and global levels. For example, in January 2020, the WHO published its first COVID-19 course through its OpenWHO platform. According to the weekly operational update on COVID-19 published by the WHO, more than 6 million people had enrolled on 39 courses, and more than 3.2 million certificates had been issued by the platform [6]. The purpose of these courses is to improve the response to health emergencies and obtain the latest scientific and operational know-how. Although many of these courses are directed to the public, some are directed to health-care educators and professionals [6].

Regulatory and nursing education accreditation bodies such as the Commission on Collegiate Nursing Education [7], the Accreditation Commission for Education in Nursing [8], and the Canadian Association of Schools of Nursing [9] have issued their statements concerning the required changes to nursing education programs to adapt to the pandemic situation. These changes have stressed the concept of flexibility along with maintaining program outcomes and safety for all educational process stakeholders. Effective clinical teaching (ECT) within the pandemic context became a core concept to maintaining nursing program outcomes and safety [3–5]. The preliminary research on COVID-19 has shown that health-care professionals, including nurses, are putting their efforts into fighting the pandemic with less consciousness of their personal safety [10]. Indeed, one initial report of 44,672 COVID-19 cases from China showed a prevalence rate of 3.8% among health-care professionals [10].

In clinical teaching, the development of professional identity and expansion of clinical skills is offered through appropriate clinical learning and conducive clinical environment where theoretical knowledge is applied into the practical aspects [11]. Wider exposures in the clinical units are key factors for developing the cognitive and affective activities including the psychomotor mastery of skills [12]. The emergence of the pandemic has put the clinical nursing practice at crossroads leaving behind the concern of how to teach the clinical courses in an environment of social distancing and quarantine measures [3]. Many factors contribute to shape an effective clinical instructor such as sound preparation, awareness of attributes needed for a clinical teacher, integrating evidence-based knowledge into teaching, enhancing good communication skills, and adapting to different environmental factors. These are major components required for an ECT [13]. COVID-19 pandemic has left behind a lesson about why it is important to future-proof the clinical learning for nursing students and has highlighted the need to recalibrate the organization and facilitation of clinical education and thus produce competent and confident nurses [14]. In addition to it, the pandemic has also shown how important it is to integrate technology for clinical training and develop a variety of teaching methods to continue the clinical education. The situation has demanded the clinical trainers to be committed and develop teaching practices that would allow application of critical thinking. Trainers are expected to leave their comfort zones and have readiness to face scenarios that constantly keep changing. Updating the teaching skills is a good practice to achieve positive clinical learning outcomes [15]. Perceived characteristics for clinical instructors that are considered best are possessing a thorough knowledge and refined skills, good communication styles, being compassionate and respectful. Competent and professional clinical teachers also exhibit humanistic behaviors [13]. Awareness of those characteristics creates a path for reinforcing, modifying, and developing teaching strategies and attitudes, which in turn promotes clinical learning making it a worthy enjoyable experience [16].

In Oman, there are four nursing programs that offer a bachelor’s degree of nursing and only one (College of Nursing at Sultan Qaboos University) that offers three masters of nursing programs, and one program that offers a one-year postgraduate nursing specialized diploma (the Higher Institute of Health Specialties). Nurse educators in these programs have been largely affected by the pandemic and have created their own innovative methods to maintain ECT for their nursing students. Assessing ECT within the remote teaching situation is critical to maintaining an optimal learning experience for students. No previous studies have been conducted to assess ECT in Oman. Hence, this study aimed to assess ECT and its associated factors from nurse educators’ perspectives during the remote teaching that followed the COVID-19 pandemic.

## Methods

This study was a national web-based descriptive study. The study involved data collection using an online survey. For the purpose of this study, “nurse educator” was defined as a professional nurse engaged in teaching activities for nursing students at the time of the study or who has been engaged in teaching activities within the last 6 months.

### Settings and sampling

The study participants were recruited from four major colleges in Oman that offer a bachelor’s degree in nursing. Two of the study settings were governmental colleges, whereas the other two were private colleges. The two governmental colleges are the largest in the country. All the colleges are offering the bachelor’s degree of nursing science. Only the college affiliated to the principal investigator is offering the master’s degree of nursing science. This study utilized the convenient sampling technique. Faculty members in the study settings were invited to participate in the study based on individual invitation through their official emails. The study hyperlink was initially sent to about 400 nurse educators. A reminder was sent after 1 week from the initial invitation. The electronic survey was designed to be completed only once to prevent possible duplication of responses. No specific exclusion criteria were applied in the study. Ethical approval was secured from an affiliated university of the principal investigator as well as from the Ministry of Health research ethics committee. The survey was anonymous and no participant’s identification data were collected. On the front page of the survey, participants were given a full explanation about the study and asked to voluntarily click on the “starting” icon to commence the survey.

The current study was conducted during the initial period of the pandemic, in which the bedside-clinical teaching was suspended and replaced by online clinical teaching sessions. Data collection started on August 2020 and was finalized in December 2020. In the current study setting, the online-clinical sessions included different approaches such as online-case study discussions, watching clinical-based videos followed by online discussion, and reviewing de-identified clinical cases as well as developing an appropriate care plan. For most of the above-mentioned teaching approaches, the clinical educators were interacting with the students using both synchronous and asynchronous online modes. In the current study setting, the clinical educators were responsible for supervising a number of students ranging from 2 to 10 students, where the number of the students is reduced in the introductory clinical courses. After resuming the face-to-face clinical teaching, students were given a refreshing period where the clinical components during the lockdown period were reviewed before introducing the new clinical components.

### Sample size calculation

For the current study, a ratio of 1:10 and 1:15 subject for each predictor was used based on sample size recommendations by different researchers [17–19]. Accordingly, with a moderate effect size (R^2^ = 0.13) and a power of 0.80 and α = 0.05, and 10 predictors, the required sample size was 119 subjects. Given the fact that 127 nurse educators have filled the survey, the sample size in the current study was considered adequate for performing multiple linear regression analysis.

### Study instrument

The Effective Clinical Teaching Inventory (ECTI) was developed to meet the objective of the study. This newly developed tool was mainly based on the previous Effective Clinical Instructor Characteristics Inventory [20] and the Preceptor’s Clinical Teaching Learning Inventory [21]. Essential modifications were made based on current literature to be more relevant to major concepts of clinical teaching adhering to COVID-19 prevention and control protocols. The instrument items were stated to reflect the extent to which nurse educators were ready to conduct clinical teaching, taking into consideration the pandemic requirement. Content validity was established by obtaining expert opinion from six clinical educators (3 PhD and 3 MSN) currently in the field of clinical teaching. For the purpose of the current study, clinical educators are PhD or Master-prepared nurses who are engaged in teaching clinical courses at the time of conducting the study. In the current study setting, MSN-prepared nurse educators are primary responsible for direct supervising of students in the bedside clinical settings. Whereas PhD-prepared nurse educators are responsible for clinical course development and participating in students’ evaluation based on scheduled clinical site visits. The final version of the inventory was composed of 17 items scored on a four-point Likert scale that ranged from four (to a greater extent) to one (to a lesser extent). The items were categorized into four main dimensions including: support (5 items), professional qualities (5 items), safety (3 items), and feedback (4 items). The categorization of the tool was done based on the agreement and expertise of the research team. The tool was pilot tested among five clinical nurse educators who approved the final version for clarity and readability. All of the clinical nurse educators participated in the pilot testing were Master-prepared nurse. Along with the ECTI, biographical information of the participants was collected via a biographical information sheet. Based on the current study sample, the final modified version had a Cronbach’s alpha reliability coefficient of 0.95, which demonstrates an excellent level of internal consistency. The Cronbach’s alpha for the subscales were 0.86, 0.85, 0.82, and 0.89 for support, professional qualities, safety, and feedback subscales, respectively (see Supplement I for the complete list of the items).

The total score of the tool is ranging between 17 to 68. For the current study, the total score was categorized into 3 categories: (a) total scores less than the 50th percentile (range 17 to 43) were classified as “suboptimal effective”; (b) scores between the 50th and 75th percentiles (range 44 to 56) were classified as “satisfactory effective”; and (c) scores exceeding the 75th percentile (range 57 to 68) were classified as “highly effective”. The categorization was validated by 3 PhD-prepared nursing educators.

### Data analysis

Data were analyzed using SPSS®-PC Version 23. Descriptive statistics, including frequencies, percentages, means, and standard deviations were used to describe the study sample. The mean score of ECT was compared across different variables using an independent t-test and one-way ANOVA (with post-hoc correction where applicable), as appropriate. The normality assumption was validated before running the analysis and no serious violation was found. In order to compare the scores at the subscale level, a mean score out of four was computed for each subscale. Finally, multiple linear regression analysis was used to test possible predictors of “ECT”. For the regression modeling, we have used the “enter” method for selecting the predictors in the final model. No missing data were found for any of the study variables.

## Results

### Sample characteristics

A total of 127 nursing educators completed the electronic survey, with an overall response rate of 31.8%. The mean age of participants was 43.9 (SD = 6.9) years with a range of 30–60 years. The majority of participants (78%, *n* = 99) were female, had academic experience of more than 10 years (70.1%, *n* = 89), had a master’s degree (68.5%, *n* = 87), and a role in the training process in clinical settings (76.4%, *n* = 97). The majority of participants (78%, *n* = 99) had previous training related to infection control. However, 41.7% (*n* = 53) reported that they did not receive any training concerning teaching methods during the COVID-19 pandemic. The WHO website was the most common first choice source of information for participants (39.4%, *n* = 50) (Table 1).

**Table 1:** Sample characteristics of the nurse educators, *n* = 127

Characteristic	Number of Nurse Educator (%)
**Age, mean (SD)**	43.9 (6.9)
**Gender**	
Female	99 (78)
Male	28 (22)
**Experience as academician in years**^a^	
1–5 years	12 (9.5)
6–10 years	25 (19.9)
> 10 years	89 (70.6)
**Experience as clinician in years** ^a^	
1–2 years	51 (41.8)
3–5 years	24 (19.7)
> 5 years	47 (38.5)
**Academic Level**	
Bachelor (BSN)	12 (9.5)
Master (MSN)	87 (68.5)
Doctoral	28 (22)
**Teaching Role**^b^	
Clinical instructor	97 (76.4)
Course coordinator	24 (18.9)
Preceptor	6 (4.7)
**Received trainings related to Infection Control**	
Yes	80 (63)
No	47 (37)
**Attended staff development programs related to teaching during COVID-19**	
Yes	74 (58.3)
No	53 (41.7)
**First source of information about COVID-19**	
World Health Organization (WHO)	50 (39.4)
Ministry of Health of Oman	36 (28.3)
Social media and news	38 (29.9)
Other sources	3 (2.4)
**Total ECT score categories**	
Sub-optimal effective	22 (17.3)
Satisfactory effective	43 (33.9)
Highly effective	62 (48.8)

^a^Missing data for clinician = 5, Academician = 1

^b^Clinical instructor: A Master-prepared nurse educator who is responsible for direct supervision of the students in the clinical settings. Course coordinator: A PhD-prepared nurse educator who is responsible for clinical course development and participating in students’ evaluation based on scheduled clinical site visits. Preceptor: is a clinically active nurse who provides an in-depth clarification/education of specific procedures

### Factors associated with effective clinical teaching

The mean scores for the subscale out of four were (*M* = 3.31, *SD* = 0.67) for safety; (*M* = 3.25, *SD* = 0.76) for feedback; (*M* = 3.20, *SD* = 0.66) for professional qualities; and (*M* = 3.21, *SD* = 0.71) for support. The overall ECT score was 54.4 (*SD* = 10.9), which represented 80% of the highest possible score. According to the total score categories, the majority of the nurse educators (48.8%, *n* = 62) were classified as highly effective.

Female nurse educators reported a greater ECT level (*M* = 58.0, *SD* = 9.5) than males (*M* = 53.4, *SD* = 11.0, *p* = 0.046). The total ECT score was statistically significant across different academic levels (*F* = 6.60, *p* = 0.002). The Tukey post hoc comparison showed that doctoral prepared nurse educators reported higher ECT (*M* = 56.0, *SD* = 8.4) than masters prepared nurse educators (*M* = 55.3, *SD* = 10.1, *p* = 0.002) and bachelor prepared nurse educators (*M* = 44.1, *SD* = 15.6, *p* = 0.003). Further, masters prepared nurse educators reported a higher ECT score (*M* = 55.3, *SD* = 10.1) than bachelor prepared nurse educators (*M* = 44.1, *SD* = 15.6, *p* = 0.002).

The overall ECT score was statistically different across different teaching roles (*F* = 3.40, *p* = 0.033). Post hoc analysis with unequal variance was assumed (Tamhane procedure) and revealed that preceptors reported higher ECT score (*M* = 62.7, *SD* = 4.6), than clinical instructor (*M* = 53.2, *SD* = 11.4, *p* = 0.005). No other significant differences were detected. Moreover, nurse educators who acted as preceptors, reported a higher ECT (*M* = 62.7, *SD* = 4.6) than course coordinators (*M* = 57.7, *SD* = 8.0) and clinical instructors (*M* = 53.2, *SD* = 1.4, *p* = 0.033) (Table 2).

**Table 2:** Effective Clinical Training, mean scores and the associated factors

Characteristic	Effective Clinical Teaching M (SD)	T /F (***df***)	***p***-value
**Gender**			
Female	58.0 (9.5)	2.0 (125)	0.04
Male	53.4 (11.0)		
**Experience as academician (years)**			
1–5 years	57.6 (6.7)	1.3 (2)	0.30
6–10 years	51.86 (14.1)		
> 10 years	54.6 (10.8)		
**Experience as clinician (years)**			
1–2 years	53.8 (10.1)	0.4 (2)	0.70
3–5 years	55.9 (7.8)		
> 5 years	55.29 (12.6)		
**Academic Level**			
Bachelor (BSN)	44.1 (15.6)	6.6 (2)	0.02*
Master (MSN)	55.3 (10.1)		
Doctoral	56.0 (8.4)		
**Teaching Role**			
Clinical instructor	53.2 (11.4)	3.5 (2)	0.03^#^
Course coordinator	57.57 (8.0)		
Preceptor	62.7 (4.6)		
**Received trainings related to Infection Control**			
Yes	56.80 (7.9)	3.3 (125)	0.001
No	50.40 (11.5)		
**Attended staff development programs related to teaching during COVID-19**			
Yes	56.0 (11.4)	1.9 (125)	0.06
No	52.3 (9.7)		
**First source of information about COVID-19**			
World Health Organization (WHO)	54.3 (10.0)	0.2	0.90
Ministry of Health of Oman	53.6 (13.2)		
Social media and news	55.4 (9.9)		
Other sources	54.7 (7.0)		

^#^Tamhane post-hoc procedure (preceptors vs clinical instructors, *p* = 0.005)

*Tukey post hoc (doctoral vs masters prepared nurse educators, *p* = 0.002; doctoral vs bachelor prepared nurse educators, *p* = 0.003; masters vs bachelor prepared nurse educators, *p* = 0.002)

### Predictors of effective clinical teaching

A linear regression analysis was conducted to identify possible predictors of ECT. Variables entered in the model were age, gender, teaching role, academic level, experience as an academic, experience as a clinician, attending infection control training, attending staff development related to teaching during the COVID-19 pandemic, and first sources of information. The results revealed that age, gender, and attending infection control training were significant predictors of ECT (Table 3). The overall model was statistically significant (F = 3.00, *p* = 0.004, with an overall R = 0.45, R^2^ = 0.20, and adjusted R^2^ = 0.14).

**Table 3:** Linear Regression for Predictors of Effective Clinical Teaching

	Unstandardized Coefficients		Standardized Coefficients	***P-***value
	B	Std. Error	Beta	
(Constant)	35.15	9.70		< 0.001
Age	0.50	0.23	0.32	0.032
Gender	−5.40	2.40	−0.21	0.028
Teaching role	3.04	1.80	0.16	0.098
Academic level	2.07	1.97	0.102	0.300
Experience as academician	−0.37	0.22	−0.24	0.100
Experience as clinician	−0.15	0.22	−0.075	0.510
Attended infection control training	4.29	2.07	0.19	0.041
Attended staff development on teaching during COVID-19	1.85	1.94	0.090	0.340
Firs source of information	0.87	1.06	0.072	0.420

reference group for the Dummy variables were as the following: male (gender), clinical instructor (teaching role), BSN (academic level), No (attended infection control training), No (attended staff development), and other sources (first source of information)

## Discussion

This study aimed to assess the ECT from the nurse educators’ perspective. Gender and academic degree were identified as significantly important factors associated with different levels of ECT in that female and doctoral prepared educators were found to have higher levels of ECT. In addition, age, gender, and attending infection control training were identified as significant predictors of ECT. Clinical preceptors (bedside nurses), who acted as nurse educators reported higher ECT compared to full time faculty clinical instructors and nurse educators who acted as course coordinators. The most commonly reported source of information for participants was the formal WHO website. This finding suggests that academic staff are genuinely updated on infection control with ongoing WHO reports, guidelines, and recommendations related to the pandemic. However, we can hardly make conclusions as this could possibly be associated with the current situation of COVID-19, and not necessarily a common practice among nurse educators.

Although the current study revealed an acceptable level of ECT, about half of the participants (41.8%) reported that they did not receive any training concerning teaching methods adopted during the COVID-19 pandemic, including the use of electronic platforms and the range of options available within these platforms such as the use of shared material, the whiteboard, or the use of dynamic teaching material. In addition, knowledge about the COVID-19 pandemic and how to manage patients with this infection were not addressed nor had the educators received any training in relation to this. Although clinical teaching is the core component of the nursing curriculum, the alarming pandemic rates brought uncertainty to clinical teaching, weighing the safety of patients, students, and faculty, which demanded essential modification in clinical teaching to ensure safety of stakeholders involved in the clinical teaching process. Indeed, a substantial motion has emerged to redefine clinical teaching and how this core component of health-care education can be conducted and evaluated [3–5]. With the paradigm shift in clinical teaching during this pandemic, the role of restorative supervision and professional resilience enhancement programs became essential for ECT [22].

This study is probably one of the earliest studies focusing on the concept of ECT during the COVID-19 pandemic. The traditional view of ECT within nursing is mainly focused on nurse educators’ behaviors and their professional qualities that promote effective transfer of clinical knowledge to students [23–25]. During the current period of widespread COVID-19, this concept has become even more important for all stakeholders of the clinical teaching process [3, 26]. In the current study, among different dimensions of ECT, nurse educators in Oman reported the highest score on the safety dimension of the ECT. This reflects that the nurse educators are valuing safety among the highest priorities during bedside clinical teaching. However, the data collected in this study are limited and we could not determine the level of knowledge about COVID-19 among the educators.

The pandemic has imposed many changes to healthcare and nursing curricula. Shifting to remote and online teaching has created a new dimension to the concept of ECT [27]. Before the pandemic, the nursing curricula were based on direct supervision of the students, especially for the clinical courses. Whereas, after the pandemic different new approaches became essential competent of the nursing curricula. For example, electronic platforms to explain theoretical material, the use of educational videos, virtual simulation, and other innovative methods were used as alternative methods over traditional clinical teaching [28, 29]. Although these methods have been used in the past, the intensity of their use was much less than the current situation. This extensive use was not accompanied by appropriate assessment of the ability of health-care educators to deliver ECT. Therefore, future research should consider exploring the effects of these innovative approaches on the ECT. In addition, there were no reports on training of educators on the use of these methods to cover all clinical course objectives. Hence, future research may consider assessing the readiness of nurse educators to integrate the new innovative approaches in the clinical teaching modalities. Furthermore, reports showed that nursing students have become more independent self-leaners [30]. Students become less dependent on their nurse educators on finding the information and switch to unclassical sources of information even when it is less reliable sources [31]. Future research may consider exploring the role of nurse educators in supporting the self-learning in the ECT.

During the COVID-19 pandemic, many changes and challenges were introduced to academia and have surely influenced nursing students’ psychological status. Students have reported a high level of anxiety and stress related to the shift in their teaching methods as well as lockdown consequences [32–34]. With this stressful learning environment, students need educators who are available for support. In the current study, about 86% of nurse educators reported that they could provide the appropriate psychological support to students during their clinical teaching. However, no specific elements of this support were assessed during this study. Different reports have shown that psychological support from different parties, including educators, is a critical element to overcome students’ psychological distress associated with COVID-19 consequences, including stress associated with graduation and joining the workforce to care for patients with COVID-19 [34–36].

The unforeseen circumstances of COVID-19 has raised questions concerning the current practices of clinical training during clinical placement, whether students could go to the setting or be trained using electronic platforms [37]. In addition, other questions have been raised regarding whether clinical training would be the same after COVID-19 has been controlled or not. Therefore, it is important for nursing programs to gain insight into these perceived feelings within nurse educators and to assess areas that require optimizing, especially in clinical training strategies [3]. With this uncertainty, students need a role model to follow and guide them in this difficult leaning environment. Professional qualities of nurse educators are critical in creating a role model for students. In the current study, the majority of nurse educators (83%) reported that they can act as a role model for their students when providing direct patient care and demonstrating clinical skills while maintaining safety requirements. Different reports have stressed the importance of the role model during clinical education [38–40]. Students have always acknowledged the role model of the nurse educator as an effective method of transferring knowledge and clinical experience to them [38, 41]. Furthermore, the current study showed that preceptor nurse educators reported a higher level of ECT than other nursing educators. This finding is consistent with previous studies [16, 42, 43]. Preceptors are usually practicing nurses. Therefore, they are more engaged in direct patient care and are more aware of patient and professional safety requirements during this pandemic. This makes them excellent role models for students, who can be an effective solution for staff shortages especially when regular academic educators are unable to be available with students at the bedside level.

Many published studies have addressed the impact of COVID-19 on students’ safety, preparedness, and clinical placements. However, this study examined nurse educators’ preparedness and how well they are expected to perform as the pandemic exists. Staff development and continuing education are essential factors for ECT [44, 45]. The current study demonstrated that staff who have engaged in infection control educational programs reported a higher level of ECT. During this pandemic, the WHO have created different educational programs for health-care providers and educators [46]. These programs are aimed to enhance patient and professional safety. In the current study, more than half of the nurse educators reported that the WHO website is their first source of information. This reflects that the majority of nurse educators seek reliable sources of information in order to deliver ECT.

### Methodological aspects

The current study’s response rate is about 32%, which is relatively low for an academic community. The low response rate is commonly reported in online survey methodologies [47, 48]. Different reports have suggested that a response rate for online survey is normally ranged between 25 and 30% [49, 50]. Nevertheless, one possible reason for the low response rate in the current study could be the timing of the data collection, which was at the peak of the pandemic when academic institutions, in the country and globally, had shifted to remote and online teaching. Faculty members were mostly busy in shifting a lot of the educational material into online format, which may have resulted in survey fatigue and reduced the interest in taking part of this survey.

The univariate analysis showed that four factors were significantly associated with the overall ECT score for the nursing educators. However, using the multiple regression modeling, three significant predictors for ECT were identified with an overall adjusted R2 = 0.14. This is relatively low explanatory power. As discussed early, this study was conducted at the early stage of the pandemic. It seems the survey has not captured many different aspects related to ECT during the pandemic. For example, previous reports have suggested that providing different types of support is an essential component of ECT [51]. The current study did not tap many aspects and types of support by the nurse educators. Further, as the available information about COVID-19 in terms of prognosis, treatment, vaccines, and other related issues were still evolving, nurse educators may lack sufficient knowledge about the disease. However, the current study did not assess the knowledge about COVID-19 and hence knowledge was not included in the regression model.

### Limitations

As it was introduced earlier, the pandemic has forced nurse educators to utilize different teaching methods to supplement the clinical teaching. Although this study has explored the perspective of nurse educators about the ECT, it did not investigate the influence of these methods on the ECT because of the nature of the study design (cross-sectional). Hence, we recommend strongly that future studies investigate different teaching methods’ effect on the ECT to gain better insight into and understanding of the ECT.

This study focused on ECT from the nurse educator perspective. However, it is important to investigate students’ perspectives about ECT during the COVID-19 pandemic. Future research may consider comparing the perceptions of both parties (students and educators). Moreover, future research may consider conducting multi-country studies comparing and contrasting experiences in which global understanding of ECT during pandemic or disaster situations can be achieved. Furthermore, this study did not examine the psychological impact of this pandemic on nurse educators as they had to change many of their academic and clinical plans to accommodate the lockdowns associated with it. Many studies have investigated the impact of the pandemic on students; however, this study addressed nurse educators’ perspectives. Yet, the findings from the current study should be taken in the context of the convenient sampling and the relatively low response rate (32%) which may limit the generalizability of the findings. Finally, although the current study tool has achieved the initial psychometric properties (i.e. internal consistency and content validity), it requires further assessment by conducting an Exploratory Factor Analysis. Because of the relatively small sample size, assessing the dimensions of the tool using the factor analysis method was not feasible. Future research can consider a further assessment of the tool.

## Conclusion

Nursing education in Middle Eastern countries during COVID-19 has taught important lessons. Pandemic preparedness and mitigation plans need to be in place not only to meet the health care demands but also the training of students on health-care professional courses, especially nursing. Adequate measures including identification and appropriate training of clinical instructors and preceptors to meet clinical teaching demands during the pandemic will bring a positive impact to the education process. Therefore, it is imperative to emphasize the need to ensure educators’ readiness to use electronic platforms and methods of education, to reflect both knowledge and experience to students during limited clinical training opportunities. Sound implementation of educational policies in developing countries will help in responding to, coping with, and quickly recovering from future occurrences like emergency disasters or pandemics.

## Supplementary Information

**Additional file 1.**

## Data Availability

The datasets generated and/or analysed during the current study are not publicly available due [restrictions by the Research and Ethics Committee in the College of Nursing at Sultan Qaboos University to protect the participants’ privacy] but are available from the Principal Investigator (Omar Al-Rawajfah) on reasonable request.

## Abbreviations

ANOVA: Analysis of variance
COVID-19: Coronavirus disease 2019
ECT: Effective clinical teaching
ECTI: Effective clinical teaching inventory
M: Mean
WHO: World Health Organization
SD: Standard deviation
SPSS: Statistical Package for the Social Sciences
