# Supplementary material for: Predictors of effective clinical teaching – nursing educators’ perspective
Source: BMC Nurs. 2022 Mar 7;21:55. doi: 10.1186/s12912-022-00836-y (PMC8900108; doi:10.1186/s12912-022-00836-y)
Supplement: Supplementary file 1 — Additional file 1. [file 12912_2022_836_MOESM1_ESM.docx]

**The Effective Clinical Teaching Inventory (ECTI)**

| PROFESSIONAL QUALITIES | |
| --- | --- |
|  | I am well prepared to accomplish the clinical teaching objectives following COVID-19 prevention and control protocol. |
|  | I can assign my students to appropriate patient cases to meet the students’ clinical objectives observing COVID-19 prevention and control protocol |
|  | I can effectively teach my students the COVID-19 prevention and control protocols. |
|  | I can demonstrate/practice nursing skills on patients while observing COVID-19 prevention and control protocols. |
|  | I can show myself as a role model of patient care while observing COVID-19 prevention and control protocols. |
| FEEDBACK | |
|  | I can plan and implement appropriate incidental bedside teaching adhering to COVID-19 prevention and control protocols. |
|  | I can provide timely feedback to students while adhering to COVID-19 prevention and control protocols. |
|  | I can evaluate students’ clinical skills (e.g., dressing, medication administering, taking vital signs, etc.) and respond accordingly, while observing COVID-19 prevention and control protocols. |
| SUPPORT | |
|  | I can facilitate collaboration with healthcare team members to enhance the clinical learning of the students while observing COVID-19 prevention and control protocols. |
|  | I can encourage favorable attitudes of the students towards clinical learning during COVID-19. |
|  | I can ensure adequate availability of resources to follow COVID-19 prevention and control protocol (Sanitizers, masks, gloves, PPE kits) to facilitate clinical teaching. |
|  | I can provide psychological support to students to alleviate possible fear, anxiety, and uncertainty associated with clinical learning during COVID-19. |
|  | I can facilitate and support the students’ clinical judgment to identify at-risk COVID-19 patients and appropriately respond to this. |
| **SAFETY** | |
|  | I understand and teach COVID-19 quarantine guidelines to students. |
|  | I can ensure achieving educational objectives while observing COVID-19 prevention and control protocol in the skill laboratory or bedside training. |
|  | I can effectively identify student at-risk for COVID-19 and appropriately respond. |
|  | I can ensure students and others safety during clinical practice while appropriately adhering to COVID-19 prevention and control protocols |
